# Supplementary figures and images for: Enhanced recovery programmes versus conventional care in bariatric surgery: A systematic literature review and meta-analysis
Source: PLoS One. 2020 Dec 29;15(12):e0243096. doi: 10.1371/journal.pone.0243096 (PMC7771679; doi:10.1371/journal.pone.0243096)

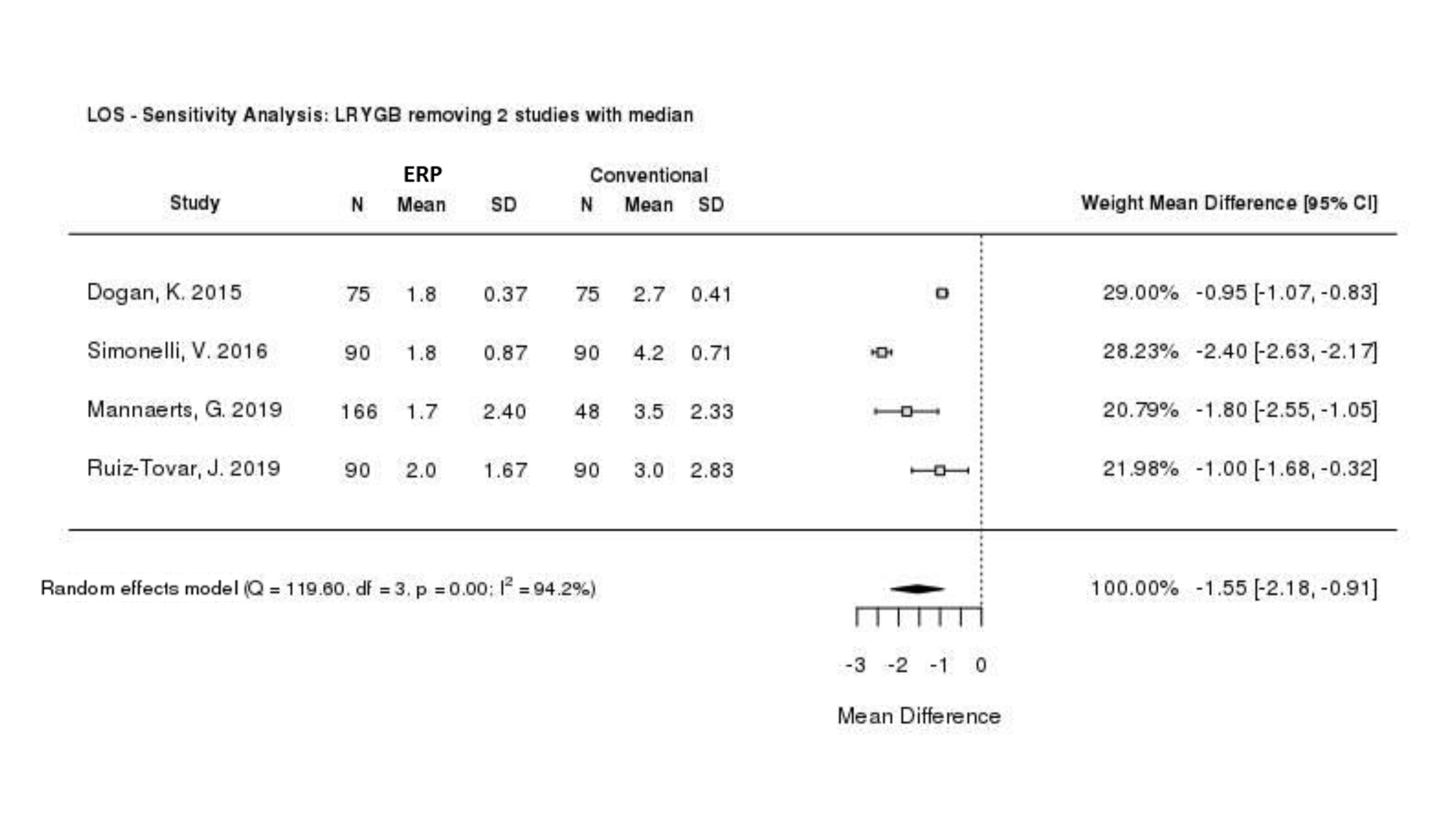

Supplement: S1 Fig — CI: Confidence interval; ERP: Enhanced recovery programme; LOS: Length of stay; RCT: Randomised controlled trial; SD: Standard deviation. (TIF) [file pone.0243096.s002.tif]

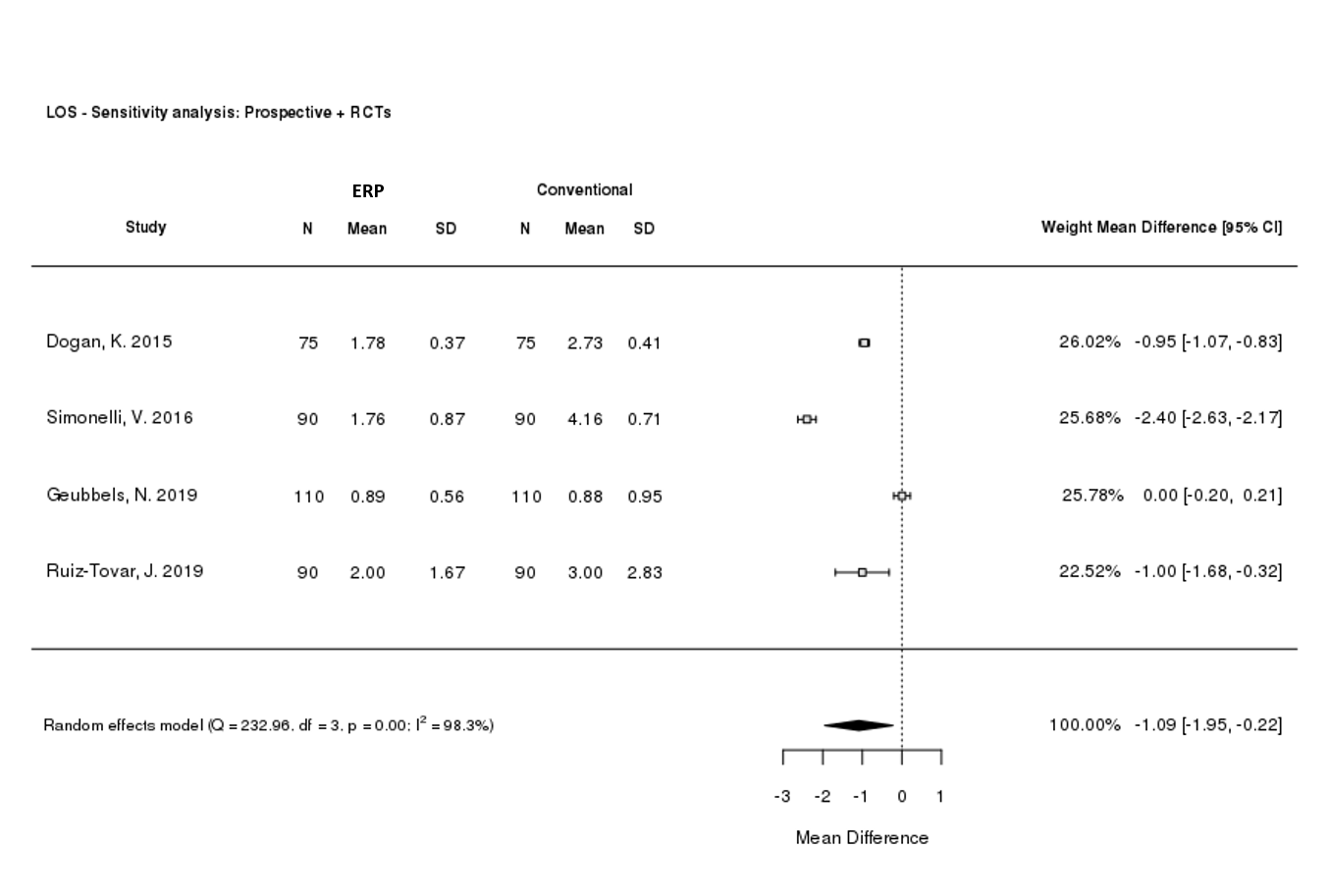

Supplement: S2 Fig — CI: Confidence interval; ERP: Enhanced recovery programme; LOS: Length of stay; RCT: Randomised controlled trial; SD: Standard deviation. (TIF) [file pone.0243096.s003.tif]

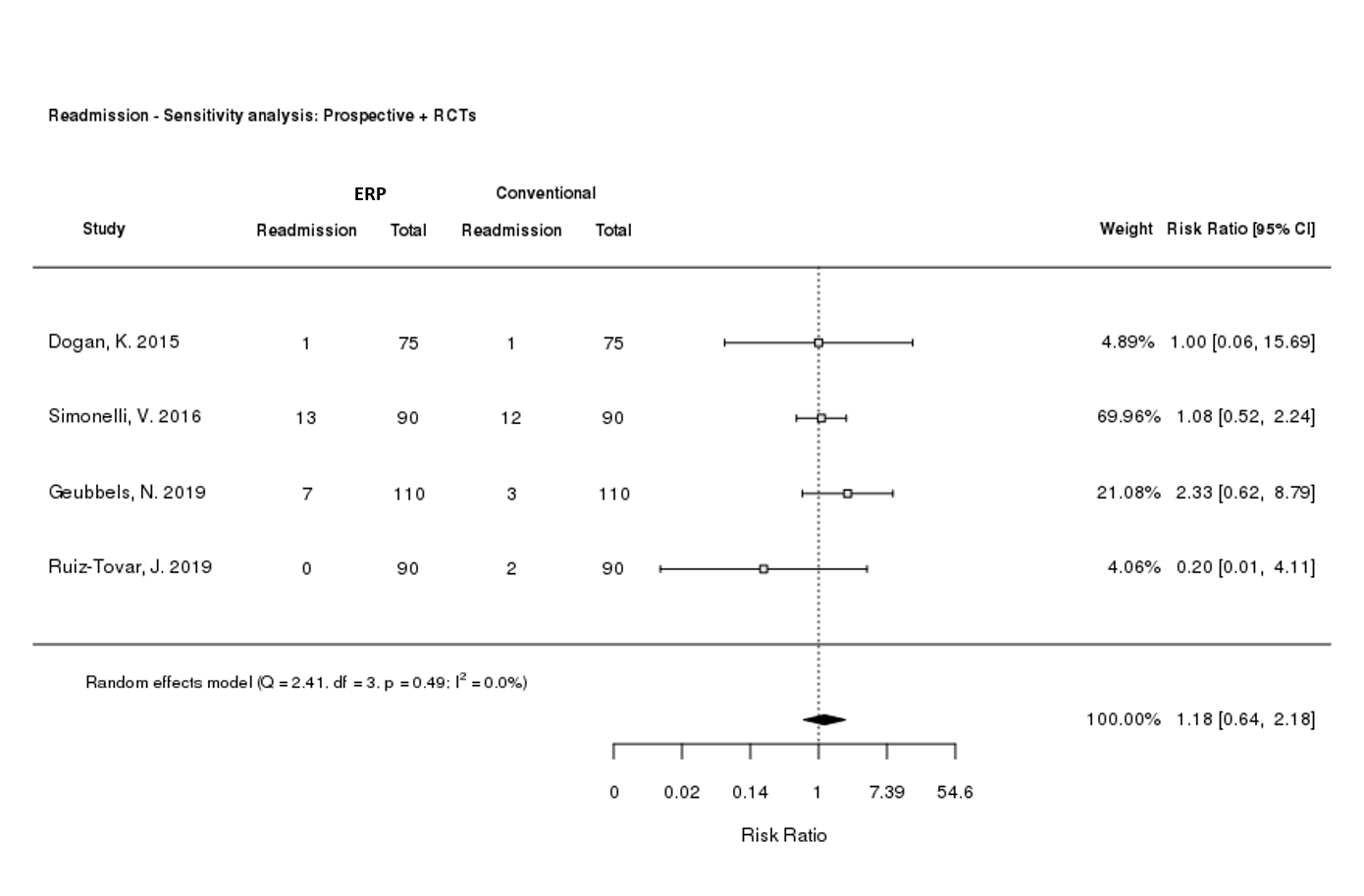

Supplement: S3 Fig — CI: Confidence interval; ERP: Enhanced recovery programme; RCT: Randomised controlled trial. (TIF) [file pone.0243096.s004.tif]
