# Supplementary material for: Enhanced recovery programmes versus conventional care in bariatric surgery: A systematic literature review and meta-analysis
Source: PLoS One. 2020 Dec 29;15(12):e0243096. doi: 10.1371/journal.pone.0243096 (PMC7771679; doi:10.1371/journal.pone.0243096)
Supplement: S2 Table — Orthopaedic search terms are included due to the intended original scope of this systematic literature review including orthopaedic surgery. (DOCX) [file pone.0243096.s006.docx]

S2 Table. Search Terms for MEDLINE, MEDLINE In-Process, MEDLINE Epub Ahead of Print and Embase – Original Review.

| **Term groups** | **#** | **Terms** | **Hits** |
| --- | --- | --- | --- |
| **Population bariatric surgery patients** | 1 | exp obesity/su | 31092 |
|  | 2 | Obes*.tw. | 613917 |
|  | 3 | exp *bariatric surgery/ | 36342 |
|  | 4 | (bariatric surgery or gastroplast* or gastric bypass* or Roux-en-Y or gastric band* or biliopancreatic diversion* or gastrectom* or duodenal switch* or gastrointestinal diversion* or gastroenterostom* or jejunoileal bypass*).tw. | 111858 |
|  | 5 | (GBP or AGB or BPD or DS or RYGB or SG).ti. | 4224 |
|  | 6 | (GBP or AGB or BPD or DS or RYGB or SG).ab. /freq=2 | 73907 |
|  | 7 | (("weight loss" or bariatric) adj2 (surger* or surgic* or procedure*)).tw. | 37658 |
|  | 8 | or/1-7 | 761605 |
| **Population: hip, knee and fracture patients** | 9 | *arthroplasty, replacement, hip/ | 20190 |
|  | 10 | hip prosthesis/ or femur implant/ | 32855 |
|  | 11 | *arthroplasty, replacement, knee/ | 18027 |
|  | 12 | knee prosthesis/ or knee implant/ | 18766 |
|  | 13 | (THA or TKA).ti. | 3665 |
|  | 14 | (THA or TKA).ab. /freq=2 | 25449 |
|  | 15 | or/9-14 | 87710 |
|  | 16 | (hip or hips or pelvi* or knee*).ti. | 348690 |
|  | 17 | (hip or hips or pelvi* or knee*).ab. /freq=2 | 393095 |
|  | 18 | hip/su | 5528 |
|  | 19 | knee/su | 6475 |
|  | 20 | or/16-19 | 549006 |
|  | 21 | exp fractures, bone/su or exp hip fractures/su or exp fracture fixation/ | 201235 |
|  | 22 | (fracture* or "non-union" or "nonunion").ti,ab. | 506344 |
|  | 23 | (surgery or surgical* or surgeries or surgeon* or procedure* or orthopaedic* or orthopedic*).ti,ab. | 553947 |
|  | 24 | 22 and 23 | 157369 |
|  | 25 | 21 or 24 | 289008 |
|  | 26 | exp joint prosthesis/ | 104796 |
|  | 27 | prosthesis implantation/ or exp arthroplasty, replacement/ | 69642 |
|  | 28 | (arthroplast* or implant* or replace* or prosthe* or endoprosthe* or surgery or surgical* or surgeries or surgeon* or procedure* or orthopaedic* or orthopedic*).ti,ab. | 6776794 |
|  | 29 | exp orthopedic manipulation/ | 6140 |
|  | 30 | or/25-29 | 6894507 |
| **Intervention** | 31 | ERAS.ti,ab. | 7256 |
|  | 32 | (fast-track adj5 (recovery or rehabilitation)).ti,ab. | 940 |
|  | 33 | (early adj2 discharge).ti,ab. | 8401 |
|  | 34 | (Fast and track and surgery).ti,ab. | 3180 |
|  | 35 | ("enhanced recovery" adj4 (protocol or pathway or program or program or surgery or multimodal or multi-modal)).ti,ab. | 3601 |
|  | 36 | or/31-35 | 20331 |
| **Exclusion terms** | 37 | Conference abstract.pt. | 2735320 |
|  | 38 | limit 37 to yr="1860 - 2014" | 1895629 |
|  | 39 | exp animals/ not exp humans/ | 9426301 |
|  | 40 | exp Comment/ or comment.pt. | 725038 |
|  | 41 | exp Editorial/ or editorial.pt. | 1056668 |
|  | 42 | exp Letter/ or letter.pt. | 2021500 |
|  | 43 | "Case reports".pt. | 1978956 |
|  | 44 | (case stud$ or case report$ or protocol$).ti. | 674455 |
|  | 45 | or/38-44 | 16475957 |
| **Total** | 46 | 30 and 20 | 278628 |
|  | 47 | 8 or 15 or 25 or 46 | 1294873 |
|  | 48 | 47 and 36 | 1513 |
|  | 49 | 48 not 45 | 1179 |
|  | 50 | limit 49 to yr="2012-2017" | 820 |
|  | 51 | remove duplicates from 50 | 505 |

Orthopaedic search terms are included due to the intended original scope of this systematic literature review including orthopaedic surgery.
