# Supplementary material for: Enhanced recovery programmes versus conventional care in bariatric surgery: A systematic literature review and meta-analysis
Source: PLoS One. 2020 Dec 29;15(12):e0243096. doi: 10.1371/journal.pone.0243096 (PMC7771679; doi:10.1371/journal.pone.0243096)
Supplement: S3 Table — (DOCX) [file pone.0243096.s007.docx]

S3 Table. Search Terms for MEDLINE, MEDLINE In-Process, MEDLINE Epub Ahead of Print and Embase – Update Review.

| **Term groups** | **#** | **Terms** | **Hits** |
| --- | --- | --- | --- |
| **Population bariatric surgery patients** | 1 | exp obesity/su | 34765 |
|  | 2 | Obes*.tw. | 683404 |
|  | 3 | exp *bariatric surgery/ | 44004 |
|  | 4 | (bariatric surgery or gastroplast* or gastric bypass* or Roux-en-Y or gastric band* or biliopancreatic diversion* or gastrectom* or duodenal switch* or gastrointestinal diversion* or gastroenterostom* or jejunoileal bypass*).tw. | 125488 |
|  | 5 | (GBP or AGB or BPD or DS or RYGB or SG).ti. | 4887 |
|  | 6 | (GBP or AGB or BPD or DS or RYGB or SG).ab. /freq=2 | 84266 |
|  | 7 | (("weight loss" or bariatric) adj2 (surger* or surgic* or procedure*)).tw. | 46639 |
|  | 8 | or/1-7 | 846403 |
| **Intervention** | 9 | ERAS.ti,ab. | 9513 |
|  | 10 | (fast-track adj5 (recovery or rehabilitation)).ti,ab. | 1009 |
|  | 11 | (early adj2 discharge).ti,ab. | 9368 |
|  | 12 | (Fast and track and surgery).ti,ab. | 3486 |
|  | 13 | ("enhanced recovery" adj4 (protocol or pathway or program or programme or surgery or multimodal or multi-modal)).ti,ab. | 5735 |
|  | 14 | or/9-13 | 24600 |
| **Exclusion terms** | 15 | Conference abstract.pt. | 3465656 |
|  | 16 | limit 15 to yr="1860 - 2014" | 1897375 |
|  | 17 | exp animals/ not exp humans/ | 9071586 |
|  | 18 | exp Comment/ or comment.pt. | 785523 |
|  | 19 | exp Editorial/ or editorial.pt. | 1162789 |
|  | 20 | exp Letter/ or letter.pt. | 2114121 |
|  | 21 | "Case reports".pt. | 2031291 |
|  | 22 | (case stud$ or case report$ or protocol$).ti. | 732701 |
|  | 23 | or/16-22 | 16439630 |
| **Total** | 24 | 8 and 14 | 838 |
|  | 25 | 24 not 23 | 651 |
|  | 26 | limit 25 to yr="2012-2019" | 540 |
|  | 27 | remove duplicates from 26 | 372 |
