# Supplementary material for: Enhanced recovery programmes versus conventional care in bariatric surgery: A systematic literature review and meta-analysis
Source: PLoS One. 2020 Dec 29;15(12):e0243096. doi: 10.1371/journal.pone.0243096 (PMC7771679; doi:10.1371/journal.pone.0243096)
Supplement: S4 Table — Orthopaedic search terms are included due to the intended original scope of this systematic literature review including orthopaedic surgery. (DOCX) [file pone.0243096.s008.docx]

S4 Table. Search Terms for the Cochrane Library Databases (Searched via the Wiley Online Platform) – Original Review.

| **Term groups** | **#** | **Terms** | **Hits** |
| --- | --- | --- | --- |
| **Population: bariatric surgery** | 1 | [mh obesity] | 10518 |
|  | 2 | Obes*:ti,ab,kw | 24073 |
|  | 3 | [mh “bariatric surgery”] | 1032 |
|  | 4 | ("bariatric surgery" or gastroplast* or "gastric bypass" * or "Roux-en-Y" or "gastric band" * or "biliopancreatic diversion" * or gastrectom* or "duodenal switch" * or "gastrointestinal diversion" * or gastroenterostom* or "jejunoileal bypass" *):ti,ab,kw | 3908 |
|  | 5 | (GBP or AGB or BPD or DS or RYGB or SG):ti | 247 |
|  | 6 | (GBP or AGB or BPD or DS or RYGB or SG):ab | 2968 |
|  | 7 | (("weight loss" or bariatric) near/2 (surger* or surgic* or procedure*)):ti,ab,kw | 1272 |
|  | 8 | OR #1-#7 | 29116 |
| **Population:**  **hip, knee and fracture patients** | 9 | [mh ^"Arthroplasty, Replacement, Hip"] | 1969 |
|  | 10 | [mh ^"hip prosthesis"] or [mh ^"femur implant"] | 1167 |
|  | 11 | [mh ^"arthroplasty, replacement, knee" [mj]] | 500 |
|  | 12 | [mh ^"knee prosthesis"] or [mh ^"knee implant"] | 715 |
|  | 13 | (THA or TKA):ti | 304 |
|  | 14 | (THA or TKA):ab | 1974 |
|  | 15 | OR #9-#14 | 5091 |
|  | 16 | (hip or hips or pelvi* or knee*):ti | 17776 |
|  | 17 | (hip or hips or pelvi* or knee*):ab | 27979 |
|  | 18 | [mh ^hip] | 376 |
|  | 19 | [mh ^knee] | 706 |
|  | 20 | OR #16-#19 | 33605 |
|  | 21 | [mh "fractures, bone"] or [mh "hip fractures"] or [mh "fracture fixation"] | 5185 |
|  | 22 | (fracture* or "non-union" or "nonunion"):ti,ab | 12722 |
|  | 23 | (surgery or surgical* or surgeries or surgeon* or procedure* or orthopaedic* or orthopedic*):ti,ab | 155791 |
|  | 24 | #22 AND #23 | 3689 |
|  | 25 | #21 OR #24 | 7333 |
|  | 26 | [mh "joint prosthesis"] | 1959 |
|  | 27 | [mh ^"prosthesis implantation"] or [mh "arthroplasty, replacement"] | 4756 |
|  | 28 | (arthroplast* or implant* or replace* or prosthe* or endoprosthe* or surgery or surgical* or surgeries or surgeon* or procedure* or orthopaedic* or orthopedic*):ti,ab | 184797 |
|  | 29 | [mh "orthopedic manipulation"] | 265 |
|  | 30 | OR #26-#29 | 188430 |
| **Intervention** | 31 | ERAS:ti,ab | 169 |
|  | 32 | ("fast-track" near/5 (recovery or rehabilitation)):ti,ab | 123 |
|  | 33 | (early NEAR/5 discharge):ti,ab | 879 |
|  | 34 | (Fast and track and surgery):ti,ab | 364 |
|  | 35 | ("enhanced recovery" NEAR/4 (protocol or pathway or program or program or surgery or multimodal or multi-modal)):ti,ab | 250 |
|  | 36 | OR #31-#35 | 1554 |
|  | 37 | [mh animals] NOT [mh humans] | 6706 |
|  | 38 | [mh Comment] or comment:pt | 1856 |
|  | 39 | [mh Editorial] or editorial:pt | 647 |
|  | 40 | [mh Letter] or letter:pt | 7686 |
|  | 41 | "Case reports":pt | 1529 |
|  | 42 | (case stud$ or case report$ or protocol$):ti | 11684 |
|  | 43 | OR #37-#42 | 28464 |
| **Total** | 44 | #20 AND #30 | 16280 |
|  | 45 | #8 OR #15 OR #25 OR #44 | 50620 |
|  | 46 | #45 AND #36 | 146 |
|  | 47 | #46 NOT #43 | 95 |

Orthopaedic search terms are included due to the intended original scope of this systematic literature review including orthopaedic surgery.
