# Supplementary material for: Enhanced recovery programmes versus conventional care in bariatric surgery: A systematic literature review and meta-analysis
Source: PLoS One. 2020 Dec 29;15(12):e0243096. doi: 10.1371/journal.pone.0243096 (PMC7771679; doi:10.1371/journal.pone.0243096)
Supplement: S5 Table — (DOCX) [file pone.0243096.s009.docx]

S5 Table. Search Terms for the Cochrane Library Databases (Searched via the Wiley Online Platform) – Update Review.

| **Term groups** | **#** | **Terms** | **Hits** |
| --- | --- | --- | --- |
| **Population: bariatric surgery** | 1 | [mh obesity] | 12258 |
|  | 2 | Obes*:ti,ab,kw | 36874 |
|  | 3 | [mh “bariatric surgery”] | 873 |
|  | 4 | ("bariatric surgery" or gastroplast* or "gastric bypass" * or "Roux-en-Y" or "gastric band" * or "biliopancreatic diversion" * or gastrectom* or "duodenal switch" * or "gastrointestinal diversion" * or gastroenterostom* or "jejunoileal bypass" *):ti,ab,kw | 63 |
|  | 5 | (GBP or AGB or BPD or DS or RYGB or SG):ti | 492 |
|  | 6 | (GBP or AGB or BPD or DS or RYGB or SG):ab | 5281 |
|  | 7 | (("weight loss" or bariatric) near/2 (surger* or surgic* or procedure*)):ti,ab,kw | 2156 |
|  | 8 | OR #1-#7 | 42427 |
| **Intervention** | 9 | ERAS:ti,ab | 490 |
|  | 10 | ("fast-track" near/5 (recovery or rehabilitation)):ti,ab | 171 |
|  | 11 | (early NEAR/5 discharge):ti,ab | 1180 |
|  | 12 | (Fast and track and surgery):ti,ab | 531 |
|  | 13 | ("enhanced recovery" NEAR/4 (protocol or pathway or program or programme or surgery or multimodal or multi-modal)):ti,ab | 570 |
|  | 14 | OR #9-#13 | 2418 |
|  | 15 | [mh animals] NOT [mh humans] | 0 |
|  | 16 | [mh Comment] or comment:pt | 1873 |
|  | 17 | [mh Editorial] or editorial:pt | 2614 |
|  | 18 | [mh Letter] or letter:pt | 11017 |
|  | 19 | "Case reports":pt | 1565 |
|  | 20 | (case stud$ or case report$ or protocol$):ti | 17457 |
|  | 21 | Or #15-#20 | 32948 |
| **Total** | 22 | #8 AND #14 | 48 |
|  | 23 | #22 NOT #21 | 42 |
