# Supplementary material for: Enhanced recovery programmes versus conventional care in bariatric surgery: A systematic literature review and meta-analysis
Source: PLoS One. 2020 Dec 29;15(12):e0243096. doi: 10.1371/journal.pone.0243096 (PMC7771679; doi:10.1371/journal.pone.0243096)
Supplement: S6 Table — ERAS: Enhanced Recovery After Surgery; ERP: Enhanced recovery programme. Orthopaedic search terms are included due to the intended original scope of this systematic literature review including orthopaedic surgery. (DOCX) [file pone.0243096.s010.docx]

S6 Table. Search Terms for Grey Literature Sources.

| **Source** | **Search Strategy** |
| --- | --- |
| **Congress Proceedings** | |
| International Society for Pharmacoeconomics and Outcomes Research Annual International Meeting | The following text strings were entered into the searches within each respective online platform:   - Fast track, fast-track - Enhanced recovery, ERP, ERAS |
| International Society for Pharmacoeconomics and Outcomes Research Annual European Congress |  |
| International Congress on Obesity |  |
| European Congress on Obesity |  |
| Congress of the European Hip Society |  |
| International Society for Technology in Arthroplasty |  |
| Orthopaedic Research Society |  |
| Groupe francophone de Réhabilitation Améliorée après Chirurgie |  |
| **Organisation Websites** | |
| European Association for the Study of Obesity website | Searches for guidelines were conducted using the following terms:   - Bariatric, hip, knee, orthopaedics, fracture, arthroplasty (for organisations specialising in ERPs) - Fast-track, fast track, enhanced recovery, ERP, ERAS (for organisations specialising in bariatric or orthopaedic surgery) |
| British Obesity & Metabolic Surgery Society website |  |
| British Orthopaedic Association website |  |
| The European Federation of National Associations of Orthopaedics and Traumatology website |  |
| ERAS® Society website |  |

ERAS: Enhanced Recovery After Surgery; ERP: enhanced recovery programme.

Orthopaedic search terms are included due to the intended original scope of this systematic literature review including orthopaedic surgery.
