# Supplementary material for: Enhanced recovery programmes versus conventional care in bariatric surgery: A systematic literature review and meta-analysis
Source: PLoS One. 2020 Dec 29;15(12):e0243096. doi: 10.1371/journal.pone.0243096 (PMC7771679; doi:10.1371/journal.pone.0243096)
Supplement: S7 Table — BMI: Body mass index; EMEA: Europe, the Middle East and Africa; EQ-5D: EuroQoL– 5 Dimensions; ERP: Enhanced recovery programme; FT: Fast track; RCT: Randomised controlled trial; SF-36: 36-Item Short Form Survey; SLR: Systematic literature review. (DOCX) [file pone.0243096.s011.docx]

S7 Table. Eligibility Criteria for the SLR.

| Category | **Inclusion criteria** | **Exclusion criteria** |
| --- | --- | --- |
| Population | - Human patients undergoing open or minimally invasive surgery within the bariatric specialty | - Animal or biomechanical studies - Studies not in a surgical, minimally invasive or endoscopic setting - Studies in which a mixed patient population is included (e.g. bariatric and colorectal surgery) without reporting data for the sub-population of interest |
| Interventions | - An ERP or other comprehensive, multi-component perioperative FT protocol | - Any study not investigating an ERP or fast-track protocol, or any study only investigating a single/several component(s) of an ERP independent of the rest of the pathway |
| Comparators | - Conventional care | - Non-comparative studies, or studies not comparing ERPs with conventional care |
| Outcomes | Studies reporting at least one of the following outcomes were included:   - Guidelines and recommendations from a formal clinical society relating to ERPs or FT protocols - Weight loss (%) - BMI reduction (%) - Diabetes resolution - EQ-5D total score - SF-36 total score - Incidence of global postoperative pain - Preoperative and postoperative anxiety - Length of stay - Readmission rate - Patient satisfaction - Total, intraoperative and postoperative mortality rates - Total, intraoperative and postoperative transfusion - Frequency of total adverse events - Incidence of thromboembolic complications - Incidence of infection (postoperative surgical site/ wound infection, respiratory infection) - Total, direct and indirect costs - Cost-effectiveness | - Studies not reporting any outcomes listed of relevance - Studies reporting relevant outcomes, but in groups of a mixed population or for a mixture of surgical procedures without reporting data for the sub-population of interest |
| Study design | - Local, national and international clinical guidance - RCTs, non-randomised interventional studies, observational studies, local/hospital registries - Economic analyses | - Case reports or case series - Letters to the editor, commentaries, seminars - Narrative reviews - National/international registries, except where is was specified that an identical ERP was used throughout |
| Other considerations | - Publications concerning patient populations, of which at least some are within the EMEA region - Published in any European language - Clinical studies in which at least some of the patients were enrolled/had surgery in 2010 or later - Non-clinical studies published in or after 2012 - Studies with ≥30 patients in any ERP arm | - Publications concerning patient populations outside of the EMEA region - Published prior to 2012 - Conference abstracts published prior to 2015 were excluded (on the assumption that any high-quality abstracts would have been published as peer-reviewed journal articles since this time) - Studies with <30 patients in all ERP arms |

BMI: body mass index; EMEA: Europe, the Middle East and Africa; EQ-5D: EuroQoL – 5 Dimensions; ERP: enhanced recovery programme; FT: fast track; RCT: randomised controlled trial; SF-36: 36-Item Short Form Survey; SLR: systematic literature review.
