# Supplementary material for: Enhanced recovery programmes versus conventional care in bariatric surgery: A systematic literature review and meta-analysis
Source: PLoS One. 2020 Dec 29;15(12):e0243096. doi: 10.1371/journal.pone.0243096 (PMC7771679; doi:10.1371/journal.pone.0243096)

S8 Table. Information Captured in the Extraction Grid.

|  | **Data or information extracted** | |
| --- | --- | --- |
| Study characteristics | - Study citation - Study design - Study location(s) - Duration of follow-up - Recruitment period - Surgical specialty and procedure | - Number of patients - Intervention and comparator - Study objective(s) - Primary study endpoint(s) - Secondary study endpoint(s) |
| Patient characteristics | - Age - Gender - BMI - Diabetes prevalence | - Insulin dependence - Use of diabetes medication - Diabetic comorbidity prevalence - ASA grade |
| Clinical and patient-reported outcomes | - Bariatric clinical outcomes: - Weight loss (%) - BMI reduction (%) - Diabetes outcomes: - Diabetes remission (%) - Diabetes improvement (%) - Diabetes-related complications (%) - Generic patient-reported outcomes - EQ-5D total score - SF-36 total score - Incidence of global postoperative pain - Preoperative and postoperative anxiety | |
| Patient recovery and operative experience outcomes | - Length of stay - Readmission rate - Patient satisfaction |  |
| Clinical safety outcomes | - Total, intraoperative and postoperative mortality rates - Total, intraoperative and postoperative transfusion - Frequency of total adverse events | - Incidence of thromboembolic complications - Incidence of infection (postoperative surgical site/ wound infection, respiratory infection) - Postoperative pain |
| Economic outcomes | - Total, direct and indirect costs | - Cost-effectiveness |
| Guidance outcomes | - Expert recommendations and national/international guidance | |

ASA: American Society of Anesthesiologists; BMI: body mass index; EQ-5D: EuroQoL – 5 Dimensions; SF-36: 36-Item Short Form Survey.


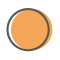

Supplement: S8 Table — ASA: American Society of Anesthesiologists; BMI: Body mass index; EQ-5D: EuroQoL– 5 Dimensions; SF-36: 36-Item Short Form Survey. (DOCX) [file pone.0243096.s012.docx]
