# Supplementary material for: Enhanced recovery programmes versus conventional care in bariatric surgery: A systematic literature review and meta-analysis
Source: PLoS One. 2020 Dec 29;15(12):e0243096. doi: 10.1371/journal.pone.0243096 (PMC7771679; doi:10.1371/journal.pone.0243096)

S9 Table. Modified Downs and Black Checklist Used for Risk of Bias Assessment of Non-randomised Studies.

| **Number** | Question | Response corresponding to lower risk of bias |
| --- | --- | --- |
| **1** | Is the hypothesis/aim/objective of the study clearly described? | Yes |
| **2** | Are the main outcomes to be measured clearly described in the Introduction or Methods section? | Yes |
| **3** | Are the characteristics of the patients included in the study clearly described? | Yes |
| **4** | Are the interventions of interest clearly described? | Yes |
| **5** | Are the distributions of principal confounders in each group of subjects to be compared clearly described? | Yes |
| **6** | Are the main findings of the study clearly described? | Yes |
| **7** | Does the study provide estimates of the random variability in the data for the main outcomes? | Yes |
| **8** | Have important adverse events that may be a consequence of the intervention been reported? | Yes |
| **9** | Have the characteristics of patients lost to follow-up been described? | Yes |
| **10** | Have actual probability values been reported (e.g. 0.035 rather than <0.05) for the main outcomes except where the probability value <0.001? | Yes |
| **11** | Were the subjects asked to participate in the study representative of the entire population from which they were recruited? | Yes |
| **12** | Were those subjects who were prepared to participate representative of the entire population from which they were recruited? | Yes |
| **13** | Were the staff, places, and facilities where the patients were treated, representative of the treatment the majority of patients receive? | Yes |
| **14** | Was an attempt made to blind study subjects to the intervention they have received? | Yes |
| **15** | Was an attempt made to blind those measuring the main outcomes of the intervention? | Yes |
| **16** | If any of the results of the study were based on “data dredging”, was this made clear? | Yes |
| **17** | In trials and cohort studies, do the analyses adjust for different lengths of follow-up of patients, or in case-control studies, is the time-period between the intervention and outcome the same for cases and controls? | Yes |
| **18** | Were the statistical tests used to assess the main outcomes appropriate? | Yes |
| **20** | Were the main outcome measures used accurate (valid and reliable)? | Yes |
| **21** | Were the patients in different intervention groups (trials and cohort studies) or were the cases and controls (case-control studies) recruited from the same population? | Yes |
| **22** | Were study subjects in different intervention groups (trials and cohort studies) or were the cases and controls (case-control studies) recruited over the same period of time? | Yes |
| **25** | Was there adequate adjustment for confounding in the analyses from which the main findings were drawn? | Yes |
| **26** | Were losses of patients to follow-up taken into account? | Yes |
| **27** | Did the study have sufficient power to detect a clinically important effect where the probability value for a difference being due to chance is less than 5%? | Yes |
| **Possible outcomes** | Yes, No, Unclear, or Not applicable | |


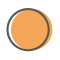

Supplement: S9 Table — (DOCX) [file pone.0243096.s013.docx]
