# Supplementary material for: Enhanced recovery programmes versus conventional care in bariatric surgery: A systematic literature review and meta-analysis
Source: PLoS One. 2020 Dec 29;15(12):e0243096. doi: 10.1371/journal.pone.0243096 (PMC7771679; doi:10.1371/journal.pone.0243096)
Supplement: S10 Table — BMI: Body mass index; CC: Conventional care; ERP: Enhanced recovery programme; NR: Not reported; SD: Standard deviation. aType 2 diabetes reported only. (DOCX) [file pone.0243096.s014.docx]

S10 Table. OAGB and LSG Data Excluded from the Analyses.

|  | **LSG – Mannaerts, G. et al, United Arab Emirates, 2019** | | **OAGB – Aktimur, R. et al, Turkey, 2018** | |
| --- | --- | --- | --- | --- |
| **Study Characteristics** | | | | |
| **Type of study** | Retrospective cohort | | Retrospective analysis of prospectively collected data | |
| **Type of care** | ERP | CC | ERP | CC |
| **Number** | 1436 | 414 | 216 | 92 |
| **Mean age (SD)** | 29.5 (9.9) | 31.0 (9.8) | 37.1 (10.5) | 38.8 (11.8) |
| **Male n (%)** | 523 (36.4) | 136 (32.9) | 57 (26.4) | 12 (13.0) |
| **Mean BMI (SD)** | 44.1 (5.6) | 45.7 (7.6) | 45.1 (7.2) | 45.1 (7.2) |
| **Comorbidities** | | | | |
| **Diabetes n (%)** | 140 (9.7) | 149 (36) | 42 (19.4)^a^ | 15 (16.3)^a^ |
| **Hypertension n (%)** | 217 (15.1) | 104 (25.1) | 62 (28.7) | 26 (28.2) |
| **Dyslipidaemia n (%)** | 208 (14) | 52 (12.6) | 26 (12.0) | 11 (11.9) |
| **Sleep apnoea n (%)** | 92 (6.4) | 111 (26.8) | 27 (12.5) | 8 (8.7) |
| **Outcomes** | | | | |
| **Mean length of hospital stay (SD)** | 1.5 (2.7) | 3.2 (7.4) | 1.2 (1.3) | 5 (0) |
| **30-day readmissions n (%)** | 38 (2.6) | 12 (2.9) | 2 (0.9) | 0 (0) |
| **30-day reoperations n (%)** | 7 (0.5) | 3 (0.7) | NR | NR |
| **30-day Clavien-Dindo grade complications n (%)** | | | | |
| **Grade I n (%)** | 198 (13.8) | 14 (3.4) | NR | NR |
| **Grade II n (%)** | 272 (18.9) | 45 (10.9) | NR | NR |
| **Grade IIIA n (%)** | 4 (0.3) | 51 (12.3) | NR | NR |
| **Grade IIIB n (%)** | 3 (0.2) | 0 (0.0) | NR | NR |
| **Grade IVA n (%)** | 4 (0.3) | 5 (1.2) | NR | NR |
| **Grade IVB n (%)** | 1 (0.1) | 1 (0.2) | NR | NR |
| **Grade V n (%)** | NR | NR | NR | NR |

BMI: body mass index; CC: conventional care; ERP: enhanced recovery programme; NR: not reported; SD: standard deviation.

^a^Type 2 diabetes reported only.
