# Supplementary material for: Enhanced recovery programmes versus conventional care in bariatric surgery: A systematic literature review and meta-analysis
Source: PLoS One. 2020 Dec 29;15(12):e0243096. doi: 10.1371/journal.pone.0243096 (PMC7771679; doi:10.1371/journal.pone.0243096)
Supplement: S11 Table — (DOCX) [file pone.0243096.s015.docx]

S11 Table. Risk of Bias Assessment Results.

| **Randomised controlled trials** | | | | |
| --- | --- | --- | --- | --- |
|  | **Geubbels 2019** | | **Ruiz-Tovar 2019** | |
| Was randomisation carried out appropriately? | Low | | Low | |
| Was the concealment of treatment allocation adequate? | High | | High | |
| Were the groups similar at the outset of the study in terms of prognostic factors? | Low | | Low | |
| Were the care providers and participants blind to treatment allocation? | High | | High | |
| Were the outcome assessors blind to treatment allocation? | High | | Low | |
| Were there any unexpected imbalances in drop-outs between groups? | Low | | Low | |
| Is there any evidence to suggest that the authors measured more outcomes than they reported? | Low | | Low | |
| Did the analysis include an intention-to-treat analysis? If so, was this appropriate and were appropriate methods used to account for missing data? | Low | | Low | |
| Are there any potential conflicts of interests (e.g. if the lead investigator(s) or design surgeons are consultants for the company, the question should be answered yes) | Low | | Low | |
| **Non-randomised studies** | | | | |
|  | **Dogan 2015** | **Geubbels 2014** | **Mannaerts 2019** | **Simonelli 2016** |
| Is the hypothesis/aim/objective of the study clearly described? | Low | Low | Low | Low |
| Are the main outcomes to be measured clearly described in the Introduction or Methods section? | Low | Low | Low | Low |
| Are the characteristics of the patients included in the study clearly described? | High | Unclear | Low | Low |
| Are the interventions of interest clearly described? | Low | Low | High | Low |
| Are the distributions of principal confounders in each group of subjects to be compared clearly described? | Low | Low | Low | Low |
| Are the main findings of the study clearly described? | High | Low | Low | Low |
| Does the study provide estimates of the random variability in the data for the main outcomes? | Low | Unclear | Low | Low |
| Have important adverse events that may be a consequence of the intervention been reported? | Low | Low | Low | Low |
| Have the characteristics of patients lost to follow-up been described? | High | Unclear | High | Low |
| Have actual probability values been reported (e.g. 0.035 rather than <0.05) for the main outcomes except where the probability value <0.001? | Low | High | Low | Low |
| Were the subjects asked to participate in the study representative of the entire population from which they were recruited? | Unclear | Low | Low | Unclear |
| Were those subjects who were prepared to participate representative of the entire population from which they were recruited? | Unclear | Unclear | Low | Unclear |
| Were the staff, places, and facilities where the patients were treated, representative of the treatment the majority of patients receive? | Unclear | Unclear | Unclear | Unclear |
| Was an attempt made to blind study subjects to the intervention they have received? | Unclear | Unclear | High | Unclear |
| Was an attempt made to blind those measuring the main outcomes of the intervention? | Unclear | Unclear | High | Unclear |
| If any of the results of the study were based on “data dredging”, was this made clear? | Low | Low | Low | Low |
| In trials and cohort studies, do the analyses adjust for different lengths of follow-up of patients, or in case-control studies, is the time-period between the intervention and outcome the same for cases and controls? | Low | Low | Unclear | Low |
| Were the statistical tests used to assess the main outcomes appropriate? | Low | Low | Low | Low |
| Were the main outcome measures used accurate (valid and reliable)? | Low | Low | Low | Low |
| Were the patients in different intervention groups (trials and cohort studies) or were the cases and controls (case-control studies) recruited from the same population? | Low | Low | Low | Low |
| Were study subjects in different intervention groups (trials and cohort studies) or were the cases and controls (case-control studies) recruited over the same period of time? | High | High | High | Low |
| Was there adequate adjustment for confounding in the analyses from which the main findings were drawn? | Unclear | Low | High | Low |
| Were losses of patients to follow-up taken into account? | Unclear | Unclear | Unclear | Unclear |
| Did the study have sufficient power to detect a clinically important effect where the probability value for a difference being due to chance is less than 5%? | Unclear | Unclear | Unclear | Unclear |
