# Supplementary material for: Enhanced recovery programmes versus conventional care in bariatric surgery: A systematic literature review and meta-analysis
Source: PLoS One. 2020 Dec 29;15(12):e0243096. doi: 10.1371/journal.pone.0243096 (PMC7771679; doi:10.1371/journal.pone.0243096)
Supplement: S12 Table — CRP: C-reactive protein; VAS: Visual analogue scale. (DOCX) [file pone.0243096.s016.docx]

S12 Table. Discharge Criteria Reported in Included Studies.

| **Study** | **Discharge criteria** |
| --- | --- |
| **Dogan 2015** | - Adequate pain control - No fever (temperature <38°C) - No tachycardia (rate <100 per minute) - Normal blood pressure (<160/90 mmHg) - Adequate oral intake |
| **Geubbels 2014** | - Well-controlled pain (VAS <4) with only aminocetaphen - No fever (>38.5 °C) - Pulse <100 bpm - No nausea/vomiting |
| **Geubbels 2019** | - Pain adequately controlled (VAS <4) with paracetamol and non-steroidal anti-inflammatory drugs - No fever - No postoperative nausea and vomiting - Patient tolerating full liquid diet - Patient able to mobilise independently - Patient feeling fit for discharge |
| **Mannaerts 2019** | Not reported |
| **Ruiz-Tovar 2019** | - Pain controlled with oral analgesia - No fever - No surgical complications - Full deambulation - Patient acceptance |
| **Simonelli 2016** | - Adequate pain control - No fever (temperature <38°C) - No tachycardia (rate <100 per minute) - Normal blood pressure (<160/90 mmHg) - Adequate oral intake - White blood cell count <17,000 mg/dl - CRP <100 |

CRP: C-reactive protein; VAS: visual analogue scale.
