# Supplementary material for: Enhanced recovery programmes versus conventional care in bariatric surgery: A systematic literature review and meta-analysis
Source: PLoS One. 2020 Dec 29;15(12):e0243096. doi: 10.1371/journal.pone.0243096 (PMC7771679; doi:10.1371/journal.pone.0243096)
Supplement: Attachment — Submitted filename: REVIEW.docx [file pone.0243096.s017.docx]

Review of the manuscript “Enhanced Recovery Programmes Versus Conventional Care in Bariatric Surgery: A Systematic Literature Review and Meta-Analysis”

Since the end of the 90s of the last century, there have been many reports describing a method defined as ERPs. The strategy of fast track gathers various elements of perioperative procedure. It takes into account the pathophysiology of operation injury and eliminates surgery procedures that are not justified in the perspective of evidence-based medicine. Optimal preparation of a patient for the operation connected with oral and written information about the surgical procedure and postoperative course, early feeding and rehabilitation on the day of surgery and optimal pain control make up the most important elements of pre- and post-operative procedures based on fast track surgery. The intraoperative factors include minimal-access surgery, thoracic epidural anesthesia and no routine use of nasogastric tube and abdominal drains. The effect of such a procedure is a decrease of postoperative complications, improvement of patient’s comfort and satisfaction, and shortening of hospital stay, and at the same time, reduction of hospitalization costs.

The authors presented systematic literature review aimed to evaluate the effects of enhanced

recovery programmes after LRYGB. The results can be drawn that using ERP in the obese patients qualified to LRYGB shortens length of hospital stay.

The study design is clear and well described. The used methods are generally acceptable.

The topic of article is interesting, methods of the study are presented concisely.

My remarks:

1. In figure 2 authors characterised of ERP implementation for included studies. Analysis of fig 2 suggest that data about important elements of ER programme are unable to determine in most analysed articles. It is difficult to compare the results precisely since the trials applied variable protocols of enhanced recovery programs and each study assumed various final points. In my opinion it should be cleary described and highlights in the manuscript.

2. Although we have an access to a few study on ERPs, it is difficult to prove, in an unambiguous manner, an advantage of one of the methods. It seems that relaying on the presented results, a conclusion can be drawn that using ERPs shortens length of hospital stay, but further RCTs study are necessary.

Authors concluded that “…results of this review support the use of ERPs in terms of faster time to discharge…”.

What was criteria of discharging a patient from hospital in analyzed study? There are same or differ in these study, it can impacts of obtained results.

3.References should be checked according to submission guidelines
