# Supplementary material for: Enhanced recovery programmes versus conventional care in bariatric surgery: A systematic literature review and meta-analysis
Source: PLoS One. 2020 Dec 29;15(12):e0243096. doi: 10.1371/journal.pone.0243096 (PMC7771679; doi:10.1371/journal.pone.0243096)
Supplement: Attachment — Submitted filename: Response to Reviewers.docx [file pone.0243096.s018.docx]

PONE-D-20-17131 - Response to Reviewers

We thank the reviewers for their helpful feedback on the manuscript. Below are the changes we have made to address their comments. Please note that line numbers refer to the ‘Track Changes’ version of the manuscript.

| Section | Reviewer | Comments | Response [Line numbers] |
| --- | --- | --- | --- |
| Methods | Reviewer 1 | The authors referenced the paper by Hozo et al. in estimating the SD from the range (Range / 6); however, they did not clarify the sample sizes to which this formula would be applied, as it was recommended by Hozo et al. in cases where N > 70. I believe the manuscript would benefit from an increased degree of clarity if the authors were to clarify this. | Thank you, we have clarified that the N>70 version was applicable to the studies included in the analysis. [L137-139] |
| Methods | Reviewer 1 | The authors state that they used the median as an estimate for mean values in some studies. It would be ideal if the authors were to specify exactly which studies used the median as well as conduct a sensitivity analysis excluding said studies. This is because the median may have been used by the authors of the original studies because of a skewed -rather than normal- distribution of data; therefore, simply replacing it by the mean may not be appropriately representative of the study’s results. This is especially likely to be an issue if included studies had a small sample size. Conducting a sensitivity analysis will likely improve the robustness of the findings and improve the overall quality of the manuscript. | Thank you for these suggestions. We have added citations for the studies where the mean and standard deviation needed to be estimated to the Methods section. A sensitivity analysis excluding these studies has also been performed and is included as Supplementary Figure 1; the results are the same as the primary analysis including all studies. [L140-141] |
| Methods | Reviewer 1 | One concern is the focus on studies conducted in Europe, the Middle East and Africa while excluding non-English studies. Due to the linguistic variation in the aforementioned regions, this may well be a significant limitation in the findings of the review (though this limitation is acknowledged by the reviewers). Had the language restriction been removed, this may have aided the authors in finding a sufficient number of studies to perform a meta-analysis for the other two procedures (though this is difficult to ascertain since the PRISMA diagram does not specify the number of studies excluded due to the English-only limitation). If it is possible to retrieve such studies, I believe this would enhance the credibility of the review. | Thank you for raising this. The review included publications in any European language rather than using an English language limit, which we have clarified in the Methods section. No articles were ultimately excluded as a result of the language limit however, which we have now mentioned in the Discussion section. [L109-114; L303-305] |
| Methods | Reviewer 1 | Though standardized checklists are often modified to fit the purposes of each individual review, it would probably be beneficial if the authors were to clarify what modifications were done to the Downs and Blacks checklist, as this may impact the quality assessment of the included studies and quell any concerns other reviewers may have. | Thank you for this suggestion. We have added more information about how the checklist was modified to the Methods section and included the modified version of the checklist as Supplementary Table 9. [L126-128] |
| Methods | Reviewer 1 | The study is otherwise well-written and structured, and the PRISMA diagram is especially well-done. | We thank the reviewer for their feedback |
| N/A | Reviewer 2 | This is a systematic review and meta-analysis in which the authors compare the efficacy of Enhanced Recovery Programmes Versus Conventional Care in Bariatric Surgery. This is considered an updated review which supports the enhanced recovery programmes which allow shorter hospital stay. Although the study is inconclusive, it enhances the future research to fill the current data gaps. The manuscript is well-written and structure and the quality of research is scientifically sound. | We thank the reviewer for their feedback |
| Abstract | Reviewer 3 | Replace (Method) with (Methods). Also, replace (papers) with (studies). | Thank you, these changes have been made [L9-10] |
| Abstract | Reviewer 3 | Insert the software used for analysis. | We have added that analyses were conducted in R [L14] |
| Abstract | Reviewer 3 | Insert the total number of included patients. | We have added this information [L15-17] |
| Abstract | Reviewer 3 | P-values should be reported. | We have added this information [L21-24] |
| Introduction | Reviewer 3 | Line 65: insert citation for the previous meta-analyses, and highlight the unique data reported in this systematic review as well as any controversies. | Thank you for this suggestion, we have added the citations to this sentence and provided the rationale for conducting a new review in the context of these previous meta-analyses. [L68-69] |
| Methods | Reviewer 3 | The databases were searched up to 2019; please consider updating it. | Thank you for this suggestion. We have conducted targeted searches over the past year and do not believe that any major new data that would change the review’s conclusions have been published since the searches were last updated. |
| Methods | Reviewer 3 | Insert the software used for analysis. | We have added that analyses were conducted in R [L136] |
| Results | Reviewer 3 | line 141: insert reference of the included studies. | We have added the citations |
| Results | Reviewer 3 | Line 174-176: mention that the sensitivity analysis did not resolve the heterogeneity. Was the subgroup analysis performed? | We have added that the high level of heterogeneity remained in the sensitivity analysis. No subgroup analyses beyond the sensitivity analyses described in the Methods section were performed, and all results of the sensitivity analyses are reported in full in the Supplementary material (Figures S1-S3). [L188] |
| Abstract | Reviewer 3 | Line 2: (it is likely the demand), Squinting modifier!! | We have removed this from the abstract [L2] |
| Abstract | Reviewer 3 | Line 4: (XX and more efficient use) & (XX and one anastomosis gastric), insert a comma before (and). | We have added commas in these places [L4-7] |
| Abstract | Reviewer 3 | Line 15: (outcomes, however as) should be (outcomes; however, as). | We have made this change [L17] |
| Abstract | Reviewer 3 | (Meta-analysis revealed), consider adding an article (The). | We have made this change [L19] |
| Introduction | Reviewer 3 | Line 48, 51, etc.: The comma before the reference number should be replaced with a dot. | We have moved all references in the manuscript to the end of the sentences they are in. |
| Results | Reviewer 4 | In figure 2 authors characterised of ERP implementation for included studies. Analysis of fig 2 suggest that data about important elements of ER programme are unable to determine in most analysed articles. It is difficult to compare the results precisely since the trials applied variable protocols of enhanced recovery programs and each study assumed various final points. In my opinion it should be cleary described and highlights in the manuscript. | We have added further details to the Results section discussing Figure 2 to highlight the difficulty of making comparisons between the studies due to the lack of clear reporting. [L170-172] |
| Conclusions | Reviewer 4 | Although we have an access to a few study on ERPs, it is difficult to prove, in an unambiguous manner, an advantage of one of the methods. It seems that relaying on the presented results, a conclusion can be drawn that using ERPs shortens length of hospital stay, but further RCTs study are necessary. Authors concluded that “…results of this review support the use of ERPs in terms of faster time to discharge…”. | We have updated the Conclusions section to reflect the fact that there remains some uncertainty in the length of stay results, and that it would be beneficial to have additional studies to support the conclusion. [L312-320] |
| Discussion | Reviewer 4 | What was criteria of discharging a patient from hospital in analyzed study? There are same or differ in these study, it can impacts of obtained results. | Although not reported in one study, the discharge criteria used in the other studies were broadly similar. This was aligned with our expectations as the focus of the analyses was on a specific surgical procedure which should have generally universal discharge criteria. We have added a statement to the discussion section mentioning this as a possible source of variation and provided the criteria that were reported in each study in the supplementary material (Table S11). [L253-256] |
| References | Reviewer 4 | References should be checked according to submission guidelines | All of the references have been checked against the submission guidance |
