# Supplementary material for: Enhanced recovery programmes versus conventional care in bariatric surgery: A systematic literature review and meta-analysis
Source: PLoS One. 2020 Dec 29;15(12):e0243096. doi: 10.1371/journal.pone.0243096 (PMC7771679; doi:10.1371/journal.pone.0243096)
Supplement: Attachment — Submitted filename: Response to Reviewers.docx [file pone.0243096.s019.docx]

PONE-D-20-17131R1 - Response to Reviewers

We thank the reviewers for their helpful feedback on the manuscript. Below are the changes we have made to address their comments. Please note that line numbers refer to the ‘Track Changes’ version of the manuscript.

| Section | Author | Comments | Suggested Response [Line numbers] |
| --- | --- | --- | --- |
| Abstract | Reviewer 1 | Remove the number of words | Thank you, this change has been made [L1] |
| Abstract | Reviewer 1 | The study objectives is different from the final conclusion; the authors aimed to evaluate the effects of enhanced recovery programmes on three types of bariatric surgeries; however, in the final conclusion they mentioned only the laparoscopic Roux-en-Y gastric bypass surgery. Even if you couldn't include other surgeries in the analysis, you have to highlight the current evidence regarding these surgeries in the conclusion, or you can adjust your objectives. | Thank you for this comment. We have adjusted the wording of the objectives to clarify the objective was to evaluate the available evidence on the effects of enhanced recovery programmes in all three procedures, and then updated the conclusions to state that the data gaps identified by the review include the lack of data for OAGB and LSG. [L5; L32] |
| Abstract | Reviewer 1 | Line 16: add "comma" after "however". | Thank you, this change has been made [L16] |
| Abstract | Reviewer 1 | Line 18: add "hospital" to be "duration of hospital-stay". | Thank you, this change has been made [L19] |
| Abstract | Reviewer 1 | Add up to 6 keywords related to your study. | These have been added after the abstract [L33-35] |
| Introduction | Reviewer 1 | Line 35: Cite each mentioned guidelines. | Thank you, we have added the missing citations [L41-42] |
| Introduction | Reviewer 1 | The authors highlighted the association between obesity and T2DM, I suggest adding a hint about the overall morbidity and mortality associated with obesity before talking about T2DM. | Thank you, we have added the following sentences before the association with T2DM is introduced: “Severe obesity can increase the risk of hypertension, hyperlipidaemia, heart disease and ischaemic stroke, as well as a number of cancers including cancer of the colon, gall bladder, rectum and liver. Obesity is also closely linked to the development of T2DM due to a progressive decrease in insulin secretion alongside a rise in insulin resistance.” [L44-48] |
| Introduction | Reviewer 1 | Line 47: I suggest adding one sentence about the impact of bariatric surgery on insulin resistance here. | Thank you for this suggestion, we have added a sentence that bariatric surgery has been shown to improve insulin resistance, referencing [Schauer et al. 2012](https://www.nejm.org/doi/10.1056/NEJMoa1200225?url_ver=Z39.88-2003&rfr_id=ori:rid:crossref.org&rfr_dat=cr_pub%20%200www.ncbi.nlm.nih.gov) [L55-56] |
| Introduction | Reviewer 1 | The authors need to clearly present their rationale to conduct this study, as the previous two meta-analysis has similar objectives with larger sample and similar findings. | We have added that the literature reviews to inform the previous meta-analyses were conducted several years ago (May 2017 and April 2016) to clarify that the rationale for our study was to review whether the evidence limitations noted in these original analyses are still present despite additional studies having since been published. [L77-78] |
| Methods | Reviewer 1 | Please cite the protocol of this study, if you registered it in PROSPERO, or published it on an online repository. | Thank you for this suggestion. Our protocol was not registered in an online repository therefore we are unable to cite it. |
| Methods | Reviewer 1 | The last update of the study search was 15 months ago, I strongly recommend updating this search and preferably include Scopus database in your search to ensure including all potential studies. | Thank you for this suggestion. We have conducted targeted searches over the past year and do not believe that any major new data that would change the review’s conclusions have been published since the searches were last updated.  While we did not search the Scopus database, we think it would be unlikely to yield additional relevant studies in this case as we searched multiple electronic databases (including CENTRAL, MEDLINE and Embase as recommended by the Cochrane Handbook for Systematic Reviews of Interventions) and performed hand searches of citations in included studies, which is the suggested use of Scopus by the Cochrane Handbook ([Section 4](https://training.cochrane.org/handbook/current/chapter-04-technical-supplement-searching-and-selecting-studies#section-1-1-4)). |
| Methods | Reviewer 1 | Did you exclude the seventh study that includes data regarding the other surgeries? If yes (I think so), you have to adjust the objectives to include only reported surgery (as I mentioned before). If no, you have to report it's data and include it in the tables of summary. Moreover, the PRISMA flow diagram should be adjusted to include only 6 studies in the final stage. The number of included studies in the PRISMA should match the exact number of studies in the summary tables and Quality assessment. | Yes, we excluded the seventh study from the analyses as it was the only study to report data for OAGB, however it was included in the review itself due to fulfilling the eligibility criteria. We have updated the PRISMA flow diagram to make this distinction clearer and now refer only to the studies that were ultimately included in the analyses as the final level of the diagram. [Fig 1]  As the seventh study was not included in the analyses, we have added the characteristics and outcomes extracted from this study together with the data from the study reporting outcomes following LSG as a separate table in the data supplement (S10 Table). |
| Methods | Reviewer 1 | The PRISMA flow diagram should be adjusted to include the reasons of exclusion of the last 11 and 4 studies. | Thank you for this suggestion, we have updated the diagram to include these reasons. [Fig 1] |
| Methods | Reviewer 1 | Do you have any explanation for using RR instead of OR in readmission? | We used RR as this measure is more easily interpretable than OR, as per the Cochrane Handbook for Systematic Reviews of Interventions ([Section 6.4.1.2](https://training.cochrane.org/handbook/current/chapter-06#section-6-4-1-2)). |
| Methods | Reviewer 1 | Consider to conduct a subgroup analysis in the LOS outcome according to the study design to solve the heterogeneity. | A subgroup analysis was conducted to explore this (please see Fig S2), however it was not found to account fully for the heterogeneity. |
| Discussion | Reviewer 1 | "This review explored the evidence available for the impact that ERPs have on outcomes in patients undergoing three common bariatric procedures". You evaluated the impact of ERPs on only one surgery not three, please correct. | The objective of the review was not purely to evaluate the impact but to examine what evidence was available to inform such an evaluation, therefore the confirmation of a lack of evidence for these procedures was part of the findings of the review. We have updated the wording of the opening sentence in the discussion to make this clearer: “This review explored what evidence was available for the impact that ERPs have on outcomes in patients undergoing three common bariatric procedures” [L237] |
| Discussion | Reviewer 1 | "As only one relevant study was identified for each of LSG and OAGB, meta-analysis was not possible, and results are therefore not  discussed for these procedures." State clearly that you exclude the study from your review, as you did not report and data regarding it. | As per the previous comment we have updated the PRISMA flow diagram to make the distinction between studies included in the review versus the analysis clearer. We have also updated the results section to refer to the data supplement where characteristics and outcomes for these studies are presented. [ L162-163] |
| Discussion | Reviewer 1 | Line 258 "reporting recent clinical and economic evidence related to ERPs in LRYGB, OAGB and LSG within EMEA" You cannot consider this as a strength point as you did not report any data regarding OAGB and LSG. | This sentence has been rephrased to clarify that the review methodology was designed to identify these data as the inability to report analyses is a product of the lack of published data rather than a strength or weakness of the review methodology. [L305] |
| Discussion | Reviewer 1 | Significant heterogeneity and the  relatively small number of studies should be added to the section of limitations. | Thank you for this suggestion, we have added further details of the heterogeneity and the small number of studies to the paragraph discussing evidence gaps. [L297-300] |
| Throughout | Reviewer 1 | The manuscript needs language editing to eliminate any mistakes. | Thank you, we have performed a full review of the manuscript and updated wording throughout. |
| Throughout | Reviewer 3 | The authors have addressed all my comments/suggestions. | Thank you very much for your review of the revised manuscript |
| Throughout | Reviewer 4 | I have no comments. I accepted author response to manuscripte Enhanced Recovery Programmes Versus Conventional Care in Bariatric Surgery: A Systematic Literature Review and Meta-Analysis | Thank you very much for your review of the revised manuscript |
